# Left atrial diastasis strain slope is a marker of hemodynamic recovery in post-ST elevation myocardial infarction: the Laser Atherectomy for STemi, Pci Analysis with Scintigraphy Study (LAST-PASS)

**DOI:** 10.3389/fradi.2024.1294398

**Published:** 2024-02-21

**Authors:** Yoko Kato, Wei Hao Lee, Makoto Natsumeda, Bharath Ambale-Venkatesh, Kensuke Takagi, Yuji Ikari, Joao A. C. Lima

**Affiliations:** ^1^ Division of Cardiology, Johns Hopkins University, Baltimore, MD, United States; ^2^ Department of Cardiology, Tokai University, Isehara, Japan; ^3^ Department of Radiology, Johns Hopkins University, Baltimore, MD, United States; ^4^ Department of Cardiology, Ogaki Municipal Hospital, Ogaki, Japan; ^5^ Department of Cardiology, National Cerebral and Cardiovascular Center, Suita, Japan

**Keywords:** left atrial diastasis strain slope (LADSS), left atrium, left atrial strain, left ventricular hemodynamics, LA–LV interdependency, post-STEMI, cardiac MRI, late gadolinium enhancement (LGE)

## Abstract

**Background:**

Left atrial (LA) mechanics are strongly linked with left ventricular (LV) filling. The LA diastasis strain slope (LADSS), which spans between the passive and active LA emptying phases, may be a key indicator of the LA–LV interplay during diastole.

**Aim:**

This study aimed to investigate the LA–LV interdependencies in post-ST elevation myocardial infarction (STEMI), with particular focus on the LADSS.

**Materials and methods:**

Patients with post-anterior STEMI who received primary percutaneous coronary intervention underwent contrast cardiac magnetic resonance imaging (MRI) during acute (5–9 days post-STEMI) and chronic (at 6 months) phases. The LADSS was categorized into three groups: Groups 1, 2, and 3 representing positive, flat, and negative slopes, respectively. Cross-sectional correlates of LADSS Group 2 or 3 compared to Group 1 were identified, adjusting for demographics, LA indices, and with or without LV indices. The associations of acute phase LADSS with the recovery of LV ejection fraction (LVEF) and scar amount were investigated.

**Results:**

Sixty-six acute phase (86.4% male, 63.1 ± 11.8 years) and 59 chronic phase cardiac MRI images were investigated. The distribution across LADSS Groups 1, 2, and 3 in the acute phase was 24.2%, 28.9%, and 47.0%, respectively, whereas in the chronic phase, it was 33.9%, 22.0%, and 44.1%, respectively. LADSS Group 3 demonstrated a higher heart rate than Group 1 in the acute phase (61.9 ± 8.7 vs. 73.5 ± 11.9 bpm, *p *< 0.01); lower LVEF (48.7 ± 8.6 vs. 41.8 ± 9.9%, *p *= 0.041) and weaker LA passive strain rate (SR) (−1.1 ± 0.4 vs. −0.7 [−1.2 to −0.6] s^−1^, *p *= 0.037) in the chronic phase. Chronic phase Group 3 exhibited weaker LA passive SR [relative risk ratio (RRR) = 8.8, *p *= 0.012] than Group 1 after adjusting for demographics and LA indices; lower LVEF (RRR = 0.85, *p *< 0.01), higher heart rate (RRR = 1.1, *p *= 0.070), and less likelihood of being male (RRR = 0.08, *p *= 0.058) after full adjustment. Acute phase LADSS Groups 2 and 3 predicted poor recovery of LVEF when adjusted for demographics and LA indices; LADSS Group 2 remained a predictor in the fully adjusted model (*β* = −5.8, *p *= 0.013).

**Conclusion:**

The LADSS serves both as a marker of current LV hemodynamics and its recovery in post-anterior STEMI. The LADSS is an important index of LA–LV interdependency during diastole.

**Clinical Trial Registration:**

https://clinicaltrials.gov/, identifier NCT03950310.

## Introduction

The left atrium (LA) is a dynamic chamber that has a pivotal role in the sequence of events that modulate left ventricular (LV) filling (1). LA function during the cardiac cycle can be divided into three phases: reservoir (inflow during ventricular systole), conduit (passive emptying during ventricular relaxation and diastasis), and booster pump (active emptying near ventricular end-diastole). These phases are influenced by LA relaxation, chamber stiffness, and contractility (2). The clinical significance of LA functional and deformation indices has been previously recognized; for example, LA reservoir strain is identified as a prognostic factor in the general population (3) and in patients with atrial fibrillation (4). LA–LV interdependence is important, as it underlies various scenarios, such as the incremental effect of LA reservoir strain in LV diastolic dysfunction categorization (5), prognostication through LA passive ejection fraction (LAEF passive) response during dobutamine stress magnetic resonance imaging (MRI) as a marker of ischemia-induced diastolic dysfunction (6), and prognostication through the LA coupling index (LACI), which is the ratio of minimum LA to maximum LV volume, determined from cardiac MRI in the general population (7).

The shape of the LA strain curve exhibits heterogeneity across cases, and the direction of the LA diastasis strain slope (LADSS), which spans between passive and active LA emptying phases (i.e., early LV diastolic), is not always flat. In this regard, in prior publications, representative LA strain curves have been heterogenous with regard to LADSS (3, 4, 8, 9). LADSS can be visually evaluated on LA cine images by observing the presence or absence of small LA size changes during diastasis; however, its clinical significance has not been investigated in detail.

LA function and deformation assessment in post-ST elevation myocardial infarction (STEMI) may provide insights into impaired LV mechanics and its hemodynamic recovery. LV diastolic dysfunction is a common feature in post-STEMI (10), and its impaired recovery (11, 12) is an independent predictor of poor prognosis in post-STEMI. LA reservoir strain, a marker of LV diastolic dysfunction (5, 13), has been proposed as a prognostic factor in post-STEMI; however, controversy regarding its dependency over and above LV dysfunction remains (14, 15).

Therefore, in the current study, we utilized a cohort of post-anterior STEMI patients who underwent contrast cardiac MRI in the acute and chronic phases to investigate the interdependencies of LA indices with LV function and scar and shed light on the importance of LA–LV interdependence as a predictor of prognosis after STEMI. We particularly focused on LADSS and aimed to identify its clinical significance by assessing its distribution, cross-sectional associates, and longitudinal associations of LADSS with LV functional recovery relative to scar amount in a cohort of post-anterior STEMI patients. An “Atlas of LA strain in STEMI cohort” was composed to longitudinally overview the LADSS in relation to LA volumetric, strain, and strain rate (SR) curves in a post-anterior STEMI cohort.

## Materials and methods

### Cohort

The Laser Atherectomy for STemi, Pci Analysis with Scintigraphy Study (LAST-PASS) is an ongoing multi-center prospective study in Japan (NCT03950310). The main objective of the LAST-PASS main study was to assess the impact of excimer laser coronary angioplasty (ELCA) application during the primary percutaneous coronary intervention (PCI) for first-episode anterior STEMI by evaluating myocardial salvage using I123-BMIPP and 99mTc-tetrofosmin myocardial scintigraphy. The inclusion criteria for the LAST-PASS were as follows: individuals experiencing first-episode anterior STEMI as suggested by electrocardiogram (EKG), with onset occurring within 6 h and indicated with primary PCI; those presenting with thrombolysis in myocardial infarction (TIMI) flow grade 0 or 1 at the initial coronary angiography; individuals aged 21 years or older at the time of providing consent; and those who agreed to participate in the study and provided written consent by themselves. The exclusion criteria were as follows: individuals presenting with cardiogenic shock at the time of visit; those with culprit lesions other than the proximal left anterior descending artery (LAD); those presenting with TIMI flow grade 2 or 3 at the initial coronary angiography; cases with a small reference vessel diameter of 2.5 mm or less; patients with a history of coronary artery bypass graft (CABG); those lacking the ability to provide consent due to mental or other reasons; and those judged inappropriate to attend the study by the physician in charge. Further details about LAST-PASS can be found on the ClinicalTrials website: https://clinicaltrials.gov/ct2/show/NCT03950310.

The LAST-PASS MRI sub-study was conducted from 25 July 2018 to 31 March 2021 in five MRI facilities that participated in the LAST-PASS main study. Each participant underwent two contrast cardiac MRI examinations, one in the acute phase (5–9 days post-index STEMI) and another in the chronic phase (at 6 months post-index STEMI), using 1.5- or 3.0-T scanners. The inclusion criteria for the MRI sub-study were participants enrolled in the main study who agreed to participate in the MRI sub-study, those without contraindications for MRI, body weight less than 120 kg, absence of claustrophobia, age 21 and older, and estimated glomerular filtration rate (eGFR) higher than 40 ml/min/1.73 m^2^. In cases where eGFR was 40 ml/min/1.73 m^2^ and lower, only non-contrast imaging was conducted. Exclusion criteria were those who denied to participate, presence of metal fragments in the body or post-implantation of electrical devices, pregnancy, atrial fibrillation, body weight over 120 kg, claustrophobia, age 20 and younger, allergy to gadolinium-based contrast agents (GBCAs), or other contraindications for MRI. The current study was conducted as an ancillary study of the LAST-PASS MRI sub-study.

### Clinical data

Clinical data of individual participants were collected and anonymized at each site. Then, the data were stored in the central data center. In cases where brain natriuretic peptide (BNP) information was not available, NT-pro BNP was utilized after conversion into BNP using the formula proposed by Mair et al. (lgBNP = 0.8 lgNT-proBNP − 0.018) (16). For the current study, the following information was obtained from the data center upon request: clinical data including demographics, cardiac risk factors, TIMI flow grade before and after the index PCI as assessed by the operator, maximum creatinine kinase-MB (CK-MB), and BNP levels at 6 months post-index STEMI.

### Cardiac MRI protocol

A standard cardiac MRI protocol, including cine and late gadolinium enhancement (LGE) sequences, was implemented using 1.5- or 3.0-T magnets at the five LAST-PASS MRI facilities. All MRI images were acquired under EKG gating at end-expiration. Participants eligible for contrast received intravenous administration of 0.15 mmol/kg gadobutrol (Gadovist, Bayer, Berlin, Germany). Two-chamber and four-chamber long-axis cine images, as well as multi-slice short-axis cine images covering the entire LV, were acquired with a 2D steady-state free precession (SSFP) sequence under retrospective gating, deriving 30 phases per cardiac cycle. Multi-slice LGE images covering the entire LV were acquired at 17 ± 3 min after contrast administration using a standard 2D inversion recovery gradient echo sequence or a segmented phase-sensitive inversion recovery (PSIR) gradient echo sequence. The detailed MRI protocol is summarized in Supplementary Material S1.

### Image analysis

**Figure 1:**
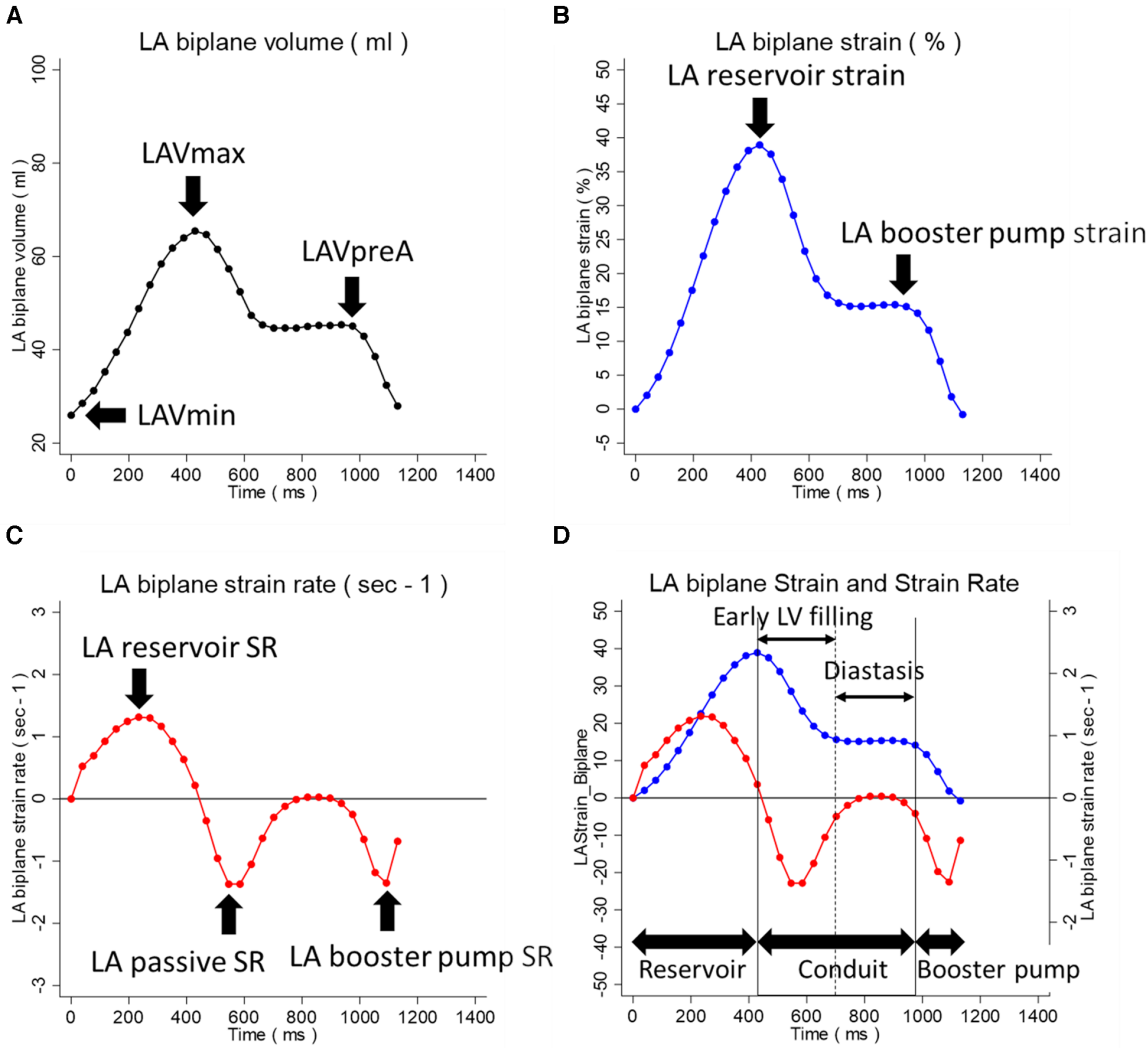
LA volumetric, strain, and strain rate indices within the LA analysis curves. (**A**) LA volumetric curve. (**B**) LA strain curve. (**C**) LA strain rate curve. (**D**) Superimposed LA strain and strain rate curves. The LA indices within the volumetric, strain, and strain rate curves are indicated. In (**D**), the three phases of LA function during the cardiac cycle, reservoir, conduit, and booster pump are indicated. The conduit phase is divided into the early LV filling and diastasis phases. LA, left atrium; LAVmax, LA maximum volume; LAVpreA, LA pre-atrial kick volume; LAVmin, LA minimum volume; LV, left ventricle.

A commercially available DICOM viewer (RadiAnt, version 2021.2; Medixant, Poznan, Poland, https://www.radiantviewer.com), and a workstation (QMass, version 7.6; Medis; Leiden, the Netherlands) were used for LV function and scar analysis. For LV function analysis, the endocardial and epicardial contours were drawn at the end-diastolic (ED) and end-systolic (ES) phases on the multi-slice short-axis cine images. Indexed LV volume at ED (LVEDVi) and ES (LVESVi) were obtained, and the LV ejection fraction (LVEF) was calculated. The scar size was analyzed on the multi-slice short-axis LGE images. The endocardial and epicardial contours were drawn, and then areas of remote myocardium and high signal intensity (SI) were automatically detected and assigned regions of interest (ROIs) by the software based on the minimum and maximum areas of SI. Scar quantification thresholding was performed with the full-width half-maximum (FWHM) technique (17, 18). The LA functional and strain analyses were performed on two-chamber and four-chamber long-axis cine images by a single observer using Multimodality Tissue Tracking software (MTT, version 6.1.4826, Toshiba, Japan). The LA volumetric measurements were automatically provided as biplane values; strain and strain rate indices were derived per plane and subsequently averaged to obtain the biplane values. The detailed analysis method is described elsewhere (8). In brief, MTT is a feature-tracking strain analysis software program, and the analyst drew the initial contour on the image at the phase with the largest LA volume. The software then propagated the contour throughout the cardiac phase, automatically tracking the LA contours. The following LA indices were collected: LA maximum volume index (LAVimax), LA pre-atrial kick volume index (LAVipreA), LA minimum volume index (LAVimin), LAEF total [=100 × (LAVmax − LAVmin)/LAVmax], LAEF passive [=100 × (LAVmax − LAVpreA)/LAVmax], LAEF booster pump [=100 × (LAVpreA − LAVmin)/LAVpreA], LA reservoir strain, LA booster pump strain, LA reservoir SR, LA passive SR, and LA booster pump SR. These indices within the volumetric, strain, and strain rate curves are indicated in Figure 1. The details of LA strain reproducibility analysis results are summarized in Supplementary Material S2. In brief, the intra-observer reproducibility of the LA strain analysis was excellent, with interclass correlation coefficients (ICCs) exceeding 0.9 for all LA strain and SR measurements.

### LADSS and the atlas of LA strain

**Figure 2:**
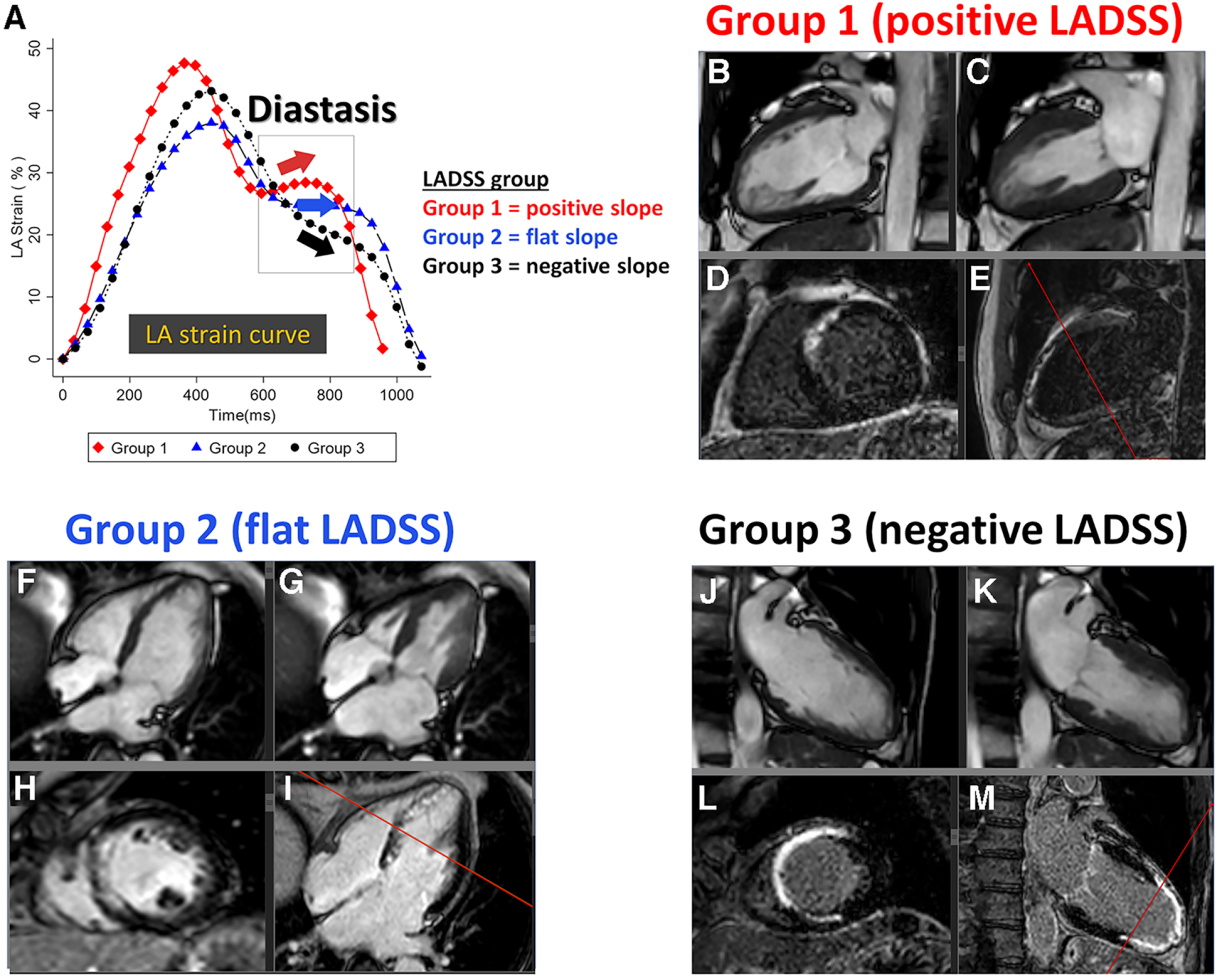
LADSS groups with representative images. (**A**) Representative LA strain curves of Groups 1, 2, and 3, reflecting a positive, flat, and negative strain slope at the diastasis phase, respectively. The individual case corresponds to each LA strain curve. LADSS Group 1: 61-year-old man, LVEF = 51.8%, LGE scar = 13.3%. LADSS Group 2: 64-year-old woman, LVEF = 50.1%, LGE scar = 11.1%. LADSS Group 3: 63-year-old man, LVEF = 30.5%, LGE scar = 21.6%. The four panels below the LADSS group name are presented in the following order: top left, two- or four-chamber cine at end-diastole (**B**, **F**, and **J**); top right, two- or four-chamber cine at end-systole (**C**, **G**, and **K**); bottom left, short-axis LGE that corresponds to the reference line in the next panel (**D**, **H**, and **L**), and bottom right, two- or four-chamber LGE (panels **E**, **I**, and **M**). The TI settings for the presented cases in Groups 1, 2, and 3 were 265, 268, and 260 ms, respectively. LADSS, left atrial diastasis strain slope; LA, left atrium; LVEF, left ventricular ejection fraction; LGE, late gadolinium enhancement; TI, inversion time.

LADSS was categorized into Groups 1, 2, and 3, representing positive, flat, and negative slopes, respectively (Figure 2). A standardized protocol to achieve consistency with visual assessment was developed to differentiate the direction of the diastasis strain slope utilizing receiver operating characteristic curve (ROC curve) analysis that considered the number of phases that take positive strain rate values during the diastasis phase against visual LADSS classification. This approach was based on the fact that the strain rate is a differential of the strain curve. Overall, LADSS Groups 1, 2, and 3 were defined as the number of positive strain rate phases during the diastasis phase being 3 and more, 1 or 2, and 0, respectively, which presented consistency with visual assessment as follows: for LADSS Group 1 (positive slope), sensitivity (Se) = 52.6%, specificity (Sp) = 97.7%, positive predictive value (PPV) = 90.9%, negative predictive value (NPV) = 82.5%, and accuracy = 84.0%; for LADSS Group 3 (negative slope), Se = 71.8%, Sp = 97.9%, PPV = 98.3%, NPV = 67.7%, and accuracy = 81.6%. The details of the LADSS group identification protocol are summarized in Supplementary Material S3. The LA volume, strain, and strain rate at both the acute and chronic phases for all cases are graphically summarized by the LADSS group in Supplementary Material S4, “Atlas of LA strain in STEMI cohort”, which provides an overview of the trends of LA strain curve shapes in each LADSS group.

### Statistical analysis

Data distribution was confirmed with histograms and Shapiro–Wilk tests. Continuous data were expressed as mean ± SD or median (first and third quartiles), depending on the distribution. Comparisons of two variables were performed using *t*-tests, paired *t*-tests, Wilcoxon rank-sum tests, or Wilcoxon signed-rank tests based on data distribution and independence. Comparisons among the three groups were performed using analysis of variance (ANOVA) or the Kruskal–Wallis test, depending on the equality of variances by Levene's test. The *post-hoc* pairwise tests were performed with Tukey's honestly significant difference (HSD) tests or Dunn's pairwise tests. The participant characteristics and MRI indices were summarized for all patients as well as by LADSS groups. The LADSS group identification protocol was developed using ROC curve analysis. The cross-sectional correlates of LADSS Groups 2 and 3 in comparison to the LADSS Group 1 were investigated using univariable and multivariable multinomial logistic regression considering demographics, heart rate, mitral regurgitation (MR), LAVimax, LA passive strain rate, LA booster pump strain rate, LVEF, and LGE scar amount. The relative risk ratios (RRRs) compared to the reference group were reported. The multivariable models were defined as follows: Model 1, the correlates of the LADSS group from demographics, heart rate, MR, and LA indices; Model 2, Model 1 + further inclusion of LVEF or LGE scar amount. The covariates in the multivariable models were stepwise forward-selected with *p *< 0.3. The chi-square probability and pseudo-*R*^2^ for the individual models were documented in the table footnotes. The associates of LADSS Group 3 against combined Groups 1 and 2 were further investigated using logistic regression by utilizing the same Models 1 and 2 including stepwise forward-selected covariates with *p *< 0.3. The longitudinal associations of the LADSS group with the recovery of LVEF and LGE scar size (ΔLVEF and ΔLGE scar amount, calculated as [the chronic phase value] − [the acute phase value]) were investigated with univariable and multivariable linear regressions. The covariates in the multivariable model were stepwise forward-selected with *p *< 0.3. The multivariable models were defined as follows: Model 1, the associations of LADSS with ΔLVEF or ΔLGE scar amount, adjusting for the demographics, heart rate, MR, and LA indices; Model 2, Model 1 + further adjustment for the counterpart LV indices (i.e., LGE scar size when the dependent variable is ΔLVEF and vice versa); Model 3, Model 2 + further adjusting for the acute phase LV index corresponding to the dependent variable and its interaction with the LADSS group. The statistical significance for LADSS groups was set at *p *< 0.017 after Bonferroni correction. The *F*-test probability and the pseudo-*R*^2^ for the individual models were documented in the table footnotes. The collinearity between the indices within the models was investigated using Pearson's correlation coefficient (*r*). When *r* presented a moderate to strong correlation, these indices were not included in the same model and these presenting differences between the LADSS groups were prioritized, e.g., LA passive SR prioritized over LA reservoir strain. STATA software (version 16.1; StataCorp; College Station, TX) was used to perform statistical calculations. The statistical significance was set at *p *< 0.05.

## Results

### Participant characteristics

Sixty-six participants underwent cardiac MRI in the acute phase (5–9 days after MI); seven of them declined to receive the chronic phase MRI. Therefore, 59 participants underwent chronic phase MRI (6 months post-MI). There were three non-contrast MRI examinations in the acute phase and six in the chronic phase. Participant characteristics are presented in Table 1. The majority of participants were men (86.4% in acute and chronic phases), the age was 63.1 ± 11.8 years in the acute phase, the maximum CK-MB was 344.5 (209–543) IU/L, and the BNP level at 6 months post-index STEMI was 61.3 (26.7–110.9) pg/ml. The heart rate was significantly reduced from the acute to chronic phases (69.8 ± 12.0 vs. 60.1 ± 8.7 bpm, *p *< 0.01). The prevalence of mitral regurgitation, pericardial effusion, and pleural effusion decreased between acute and chronic post-MI, with mitral regurgitation decreasing from 30.3% to 20.3%, pericardial effusion from 48.5% to 3.4%, and pleural effusion from 24.2% to 3.4%.

**Table 1:** Participant characteristics.

Participant characteristics	Acute phase	Chronic phase	*p-*value
*N* (%)	66 (1,000)	59 (100)	N/A
Age at MRI (years)	63.1 ± 11.8	63.5 ± 11.6	**<0.01**
Male, *N* (%)	57 (86.4)	51 (86.4)	N/A
BMI at MRI (kg/m^2^)	22.9 (21.3–24.3)	22.8 (20.9–24.2)	**0.041**
Heart rate at MRI (bpm)	69.8 ± 12.0	60.1 ± 8.7	**<0.01**
Max CK-MB (IU/L)	344.5 (209–543)	N/A	N/A
TIMI flow before index PCI			
TIMI 0	55 (84.6)	N/A	N/A
TIMI 1	10 (15.4)	N/A	N/A
TIMI flow after index PCI			
TIMI 0	1 (1.5)	N/A	N/A
TIMI 1	0 (0)	N/A	N/A
TIMI 2	1 (1.5)	N/A	N/A
TIMI 3	64 (96.7)	N/A	N/A
BNP at 6 months post-STEMI (pg/ml)	N/A	61.3 (26.7–110.9)	N/A
Smoking status			
Never	19 (28.8)	N/A	N/A
Current smoker	31 (47.0)	N/A	N/A
Past smoker	15 (22.7)	N/A	N/A
Unknown	1 (1.5)	N/A	N/A
Diabetes mellitus	13 (19.7)	N/A	N/A
Dyslipidemia	44 (66.7)	N/A	N/A
Hypertension	38 (57.6)	N/A	N/A
Mitral regurgitation present, *n* (%)	20 (30.3)	12 (20.3)	**<0.01**
Pericardial effusion present, *n* (%)	32 (48.5)	2 (3.4)	**<0.01**
Pleural effusion present, *n* (%)	16 (24.2)	2 (3.4)	**<0.01**

LADSS was grouped into Groups 1, 2, and 3, reflecting a positive, flat, and negative strain slope at the diastasis phase, respectively (Figure 2).

MRI, magnetic resonance imaging; BMI, body mass index; CK-MB, creatinine kinese-MB; TIMI, thrombolysis in myocardial infarction; PCI, percutaneous coronary intervention; BNP, brain natriuretic peptide; STEMI, ST-elevation myocardial infarction.

The bold values represent *p* < 0.05, showing statistical significance.

### Cardiac MRI indices in the acute and chronic phases post-STEMI

Table 2 summarizes the cardiac MRI indices in the acute and chronic phases. LVEF and LGE scar size changed from the acute to chronic phases [LVEF: 39.4% (32.8%–44.4%) to 44.8 ± 9.7%, LGE: 21.7 ± 12.7% to 14.1% (9.0%–21.6%), respectively, *p *< 0.01 for both]. In the LA assessment, all LA volume indices remained comparable between acute and chronic phases, while LAEF total and LAEF booster pump increased in the chronic phase compared to the acute phase [LAEF total: 53.5% (46.6%–58.3%) vs. 53.7 ± 11.5%, *p *= 0.02; LAEF booster pump: 38.6% (34.6%–44.6%) vs. 42.7% (34.4%–49.1%), *p* = 0.04]. LA reservoir strain was also higher in the chronic phase than in the acute phase (26.2 ± 8.2 vs. 29.2 ± 9.8%, *p *< 0.01). LA booster pump strain did not reach significance but presented a trend toward a higher value in the chronic phase than the acute phase.

**Table 2:** Cardiac MRI analysis results.

Cardiac MRI indices	Acute phase	Chronic phase	*p*-value
	All cases (*N* = 66)	All cases (*N* = 59)	
LV indices			
LVEDVi (ml/m^2^)	95.5 ± 18.6	93.3 (75.5–109.7)	0.53
LVESVi (ml/m^2^)	55.6 (48.7–67.0)	48.2 (38.3–66.6)	**<0**.**01**
LVMi (g/m^2^)	69.9 ± 11.8	56.7 ± 9.4	**<0**.**01**
LVEF (%)	39.4 (32.8–44.4)	44.8 ± 9.7	**<0**.**01**
LGE scar size (%)	21.7 ± 12.7	14.1 (9.0–21.6)	**<0**.**01**
LA indices			
LAVimax (ml/m^2^)	32.3 ± 8.9	32.7 ± 11.6	0.69
LAVipreA (ml/m^2^)	23.7 (19.7–31.2)	25.0 (18.6–31.1)	0.54
LAVimin (ml/m^2^)	14.4 (11.1–21.4)	13.3 (9.8–20.7)	0.27
LAEF total (%)	53.5 (46.6–58.3)	53.7 ± 11.5	**0**.**020**
LAEF passive (%)	20.6 ± 7.2	22.0 ± 7.7	0.16
LAEF booster pump (%)	38.6 (34.6–44.6)	42.7 (34.4–49.1)	**0**.**039**
LA reservoir strain (%)	26.2 ± 8.2	29.2 ± 9.8	**<0**.**01**
LA booster pump strain (%)	14.5 ± 5.7	15.8 (13.5–18.2)	0.077
LA reservoir SR (s^−1^)	1.2 ± 0.4	1.2 ± 0.4	0.62
LA passive SR (s^−1^)	−0.9 (−1.1 to −0.7)	−0.9 (−1.3 to −0.7)	0.11
LA booster pump SR (s^−1^)	−1.5 ± 0.6	−1.4 ± 0.6	0.96
LADSS group [*N* (%)]			0.45
Group 1	16 (24.2)	20 (33.9)	
Group 2	19 (28.9)	13 (22.0)	
Group 3	31 (47.0)	26 (44.1)	

LADSS was grouped into Groups 1, 2, and 3, reflecting a positive, flat, and negative strain slope at the diastasis phase, respectively (Figure 2).

MRI, magnetic resonance imaging; LV, left ventricle; LVEDVi, LV end-diastolic volume index; LVESVi, LV end-systolic volume index; LVMi, LV mass index; LVEF, LV ejection fraction; LGE, late gadolinium enhancement; LA, left atrium; LAVimax, LA maximum volume index; LAVipreA, LA pre-atrial kick volume index; LAVimin, LA minimum volume index; LAEF, LA ejection fraction; SR, strain rate; LADSS = LA diastasis strain slope.

The bold values represent *p* < 0.05, showing statistical significance.

### Demographics and cardiac MRI indices by the LADSS group

LA strain curve shapes among the different LADSS groups are shown in the “Atlas of LA strain in STEMI cohort” (Supplementary Material S4), revealing dull strain curves in Group 3 compared to Group 1. Positive, flat, and negative LADSS (i.e., Groups 1, 2, and 3) in the acute phase was observed in 24.2%, 28.9%, and 47.0% of the post-MI participants, respectively. In the chronic phase, these proportions were 33.9%, 22.0%, and 44.1%, respectively, showing an incremental proportion of positive LADSS (i.e., Group 1) but without significance (*p *= 0.45) (Table 2). The combinations of LADSS groups in the acute and chronic phases are presented in Supplementary Material S4, S5. Twenty-six cases (44.1%) maintained similar LA diastasis strain slope and were classified in the same LADSS group, while the remaining cases switched LADSS groups between the acute and chronic phases, reflecting the different direction of the strain slope during diastasis between these phases.

Demographics and cardiac MRI indices of the different LADSS groups are summarized in Supplementary Material S5. In brief, demographic data and cardiac MRI indices were generally comparable among the LADSS groups. However, for a few indices, Group 3 participants presented unfavorable alterations from a clinical viewpoint when compared to participants in Group 1, i.e., a higher heart rate in the acute phase (Group 1 vs. Group 3 = 61.9 ± 8.7 bpm vs. 73.5 ± 11.9 bpm, *p *< 0.01), a lower LVEF in the chronic phase (Group 1 vs. Group 3 = 48.7 ± 8.6% vs. 41.8 ± 9.9%, *p *= 0.041), and a weaker LA passive strain rate in the chronic phase [Group 1 vs. Group 3 = −1.1 ± 0.4 s^−1^ vs. −0.7 (−1.2 to −0.6 s^−1^), *p *= 0.037]. No significant differences were observed between participants in Group 1 vs. 2 or Group 2 vs. 3 in any of the indices. The LVEF and LGE scar size for different combinations of acute and chronic LADSS groups are summarized in a table in Supplementary Material S4. Generally, the LV function and scar size changed favorably from the acute to the chronic phase when the LADSS category improved to Group 1 or remained in the same group. Conversely, when the LADSS group transitioned toward Group 3, the LVEF and LGE scar size showed parallel changes associated with poor recovery. These trends were most evident in those participants who transitioned from Group 2 to Group 3 (LVEF: 39.3 ± 8.6% vs. 39.1 ± 9.6%, *p *= 0.94; LGE scar amount: 23.7 ± 12.9% vs. 22.3 ± 15.3%, *p *= 0.51).

### Cross-sectional correlates of LADSS groups in the acute and chronic phases

In the acute phase, a higher heart rate was correlated with LADSS Group 2 compared to Group 1 (RRR = 1.1, *p *= 0.020 in multivariable Model 2). The correlation of higher heart rate with LADSS Group 3 compared to Group 1 as a reference group was also observed in the multivariable models (RRR = 1.1, *p *= 0.019 in Model 2) (Table 3). In the chronic phase, no correlations were found for LADSS Group 2. In LADSS Group 3, in the multivariable model without LV indices (Model 1), a weaker LA passive SR was associated with LADSS Group 3, and heart rate was a marginal associate (LA passive SR: RRR = 8.8, *p *= 0.012; heart rate: RRR = 1.1, *p *= 0.057). Upon further adjustment of LV indices in Model 2, the association of LA passive SR was attenuated, and LVEF emerged as the main correlate of LADSS Group 3; female sex and higher heart rate were marginal correlates of LADSS Group 3 (LVEF: RRR = 0.85, *p *< 0.01; male sex: RRR = 0.08, *p *= 0.058; heart rate: RRR = 1.1, *p *= 0.070) (Table 4). These results were generally in line but more pronounced in the correlates of LADSS Group 3 compared to combined Groups 1 and 2 (Supplementary Material S6). In the acute phase, a higher heart rate, lower LVEF, and female sex were the correlates of LADSS Group 3 compared to combined Groups 1 and 2 in multivariable Models 1 and 2. In the chronic phase, a higher heart rate, weaker LA passive SR, and lower LVEF were correlates of LADSS Group 3 in Models 1 and 2.

**Table 3:** Cross-sectional correlates of LADSS in the acute phase.

Acute phase	Univariable		Model 1^a^		Model 2^a^	
	RRR	*p*-value	RRR	*p*-value	RRR	*p*-value
LADSS Group 1	(Reference)		(Reference)		(Reference)	
LADSS Group 2						
Age (years)	0.97	0.29	—		—	
Sex (male)	1.2	0.90	2.1	0.64	2.1	0.62
BMI (kg/m^2^)	1.2	0.19	—		—	
Heart rate (bpm)	1.1	**0**.**023**	1.1	**0**.**032**	1.1	**0**.**020**
Mitral regurgitation	1.5	0.60	—		—	
LAVimax (ml/m^2^)	0.98	0.68	—		—	
LA passive SR (s^−1^)	0.57	0.44	0.71	0.71	—	
LA booster pump SR (s^−1^)	0.62	0.45	—		—	
LVEF (%)	1.008	0.87	N/A		1.1	0.27
LGE scar amount (%)	1.03	0.34	N/A		—	
LADSS Group 3						
Age (years)	1.005	0.86	—		—	
Sex (male)	0.23	0.19	0.31	0.32	0.22	0.23
BMI (kg/m^2^)	1.1	0.55	—		—	
Heart rate (bpm)	1.1	**<0**.**01**	1.1	**<0**.**01**	1.1	**0**.**019**
Mitral regurgitation	2.7	0.17	—		—	
LAVimax (ml/m^2^)	1.03	0.36	—		—	
LA passive SR (s^−1^)	1.5	0.59	2.2	0.36	—	
LA booster pump SR (s^−1^)	0.69	0.51	—		—	
LVEF (%)	0.93	0.073	N/A		0.95	0.37
LGE scar amount (%)	1.04	0.17	N/A		—	

LADSS was grouped into Groups 1, 2, and 3, reflecting a positive, flat, and negative strain slope at the diastasis phase, respectively (Figure 2).

The chi-square probability and pseudo-*R*^2^ value of multivariable Model 1 were <0.01 and 0.13 and of Model 2 were <0.01 and 0.17, respectively.

^a^ The covariates in the models were stepwise forward-selected with *p* < 0.3. Model 1: The correlates of LADSS groups from the demographics, heart rate, presence of mitral regurgitation, and LA indices. Model 2: Model 1 + LV indices.

LADSS, left atrial diastasis strain slope; BMI, body mass index; LA, left atrium; LAVimax, maximum indexed LA volume; SR, strain rate; LVEF, left ventricular ejection fraction; LGE, late gadolinium enhancement.

The bold values represent *p* < 0.05, showing statistical significance.

**Table 4:** Cross-sectional correlates of LADSS groups in the chronic phase.

Chronic phase	Univariable		Model 1^a^		Model 2^a^	
	RRR	*p*-value	RRR	*p*-value	RRR	*p*-value
LADSS Group 1	(Reference)		(Reference)		(Reference)	
LADSS Group 2						
Age (years)	1.01	0.70	—		—	
Sex (male)	0.29	0.33	—		0.18	0.21
BMI (kg/m^2^)	1.04	0.70	—		—	
Heart rate (bpm)	1.03	0.50	1.04	0.42	1.04	0.45
Mitral regurgitation	0.73	0.74	—		—	
LAVimax (ml/m^2^)	0.995	0.88	—		—	
LA passive SR (s^−1^)	1.6	0.59	1.9	0.47	—	
LA booster pump SR (s^−1^)	1.5	0.55	—		—	
LVEF (%)	0.95	0.22	N/A		0.92	0.083
LGE scar amount (%)	1.02	0.66	N/A		—	
LADSS Group 3						
Age (years)	1.04	0.15	—		—	
Sex (male)	0.22	0.19	—		0.08	0.058
BMI (kg/m^2^)	0.96	0.68	—		—	
Heart rate (bpm)	1.1	0.087	1.1	0.057	1.1	0.070
Mitral regurgitation	1.2	0.80	—		—	
LAVimax (ml/m^2^)	1.02	0.35	—		—	
LA passive SR (s^−1^)	7.3	**0**.**018**	8.8	**0**.**012**	—	
LA booster pump SR (s^−1^)	1.4	0.56	—		—	
LVEF (%)	0.92	**0**.**021**	N/A		0.85	**<0**.**01**
LGE scar amount (%)	1.1	0.067	N/A		—	

LADSS was grouped into Groups 1, 2, and 3, reflecting a positive, flat, and negative strain slope at the diastasis phase, respectively (Figure 2). The chi-square probability and pseudo-R^2^ value of the multivariable Model 1 were 0.03 and 0.088, and, for Model 2 were <0.01 and 0.17, respectively.

^a^ The covariates in the models were stepwise forward-selected with *p* < 0.3. Model 1: The correlates of LADSS groups from the demographics, heart rate, presence of mitral regurgitation, and LA indices. Model 2: Model 1 + LV indices.

LADSS, left atrial diastasis strain slope; BMI, body mass index; LA, left atrium; LAVimax, maximum indexed LA volume; SR, strain rate; LVEF, left ventricular ejection fraction; LGE, late gadolinium enhancement.

The bold values represent *p* < 0.05, showing statistical significance.

### Association between the acute phase LADSS group and the recovery of LVEF and LGE scar amount

Table 5 presents the association between acute phase LADSS and the recovery of LVEF and changes in LGE scar amount (ΔLVEF and ΔLGE scar amount). Acute phase LADSS Group 2 was associated with poor recovery of LVEF (Model 1: *β* = −6.5, *p *< 0.01; Model 2: *β* = −6.5, *p *< 0.01). Acute phase LADSS Group 3 also showed a trend of association with poor LVEF recovery after Bonferroni correction (Model 1: *β* = −4.8, *p *= 0.04), but this trend of association was no longer observed after further adjusting for LGE scar amount in Model 2. There was no association between acute phase LADSS and ΔLGE scar in either univariable or multivariable assessments. These findings were consistent in extended Model 3, with LADSS Group 2 remaining a predictor in the fully adjusted model (*β *= −5.8, *p *= 0.013) (Supplementary Material S7).

**Table 5:** Associations of the acute phase LADSS with the recovery of LVEF and LGE scar amount.

ΔLVEF (%)^a^	Univariable		Model 1^b^		Model 2^b^	
	*β*	*p-*value	*β*	*p-*value	*β*	*p-*value
Age (years)	0.11	0.14	—	—	—	—
Sex (male)	0.66	0.80	—	—	—	—
BMI (kg/m^2^)	0.085	0.79	0.35	0.24	0.67	**0**.**043**
Heart rate (bpm)	−0.011	0.88	0.10	0.16	0.079	0.29
Mitral regurgitation	−2.7	0.14	−4.0	**0**.**040**	−4.2	**0**.**036**
LAVimax (ml/m^2^)	0.12	0.23	0.15	0.12	0.24	**0**.**023**
LA passive SR (s^−1^)	4.7	**0**.**010**	4.1	**0**.**023**	5.6	**<0**.**01**
LA booster pump SR (s^−1^)	−0.033	0.98	—	—	−2.6	0.17
LADSS^c^						
Group 1	Reference		Reference		Reference	
Group 2	−5.3	0.03	−6.5	**<0**.**01**^c^	−6.5	**<0**.**01**^c^
Group 3	−2.8	0.20	−4.8	0.04	−3.8	0.10
LGE scar (%)	−0.11	0.14	N/A	N/A	−0.11	0.13
ΔLGE scar (%)^a^	Univariable		Model 1^b^		Model 2^b^	
	*β*	*p*-value	*β*	*p*-value	*β*	*p*-value
Age (years)	0.16	0.063	0.13	0.13	0.11	0.18
Sex (male)	2.6	0.35	3.3	0.23	2.7	0.29
BMI (kg/m^2^)	−0.55	0.12	−0.57	0.11	−0.75	**0**.**032**
Heart rate (bpm)	−0.017	0.82	—	—	—	—
Mitral regurgitation	−0.51	0.81	—	—	—	—
LAVimax (ml/m^2^)	−0.14	0.18	—	—	—	—
LA passive SR (s^−1^)	1.2	0.59	—	—	—	—
LA booster pump SR (s^−1^)	−3.3	0.059	−3.2	0.067	−2.7	0.11
LADSS^c^						
Group 1	Reference		Reference		Reference	
Group 2	1.3	0.64	2.4	0.38	2.8	0.29
Group 3	−2.5	0.33	−1.1	0.67	−1.5	0.53
LVEF (%)	0.16	0.17	N/A	N/A	—	—

LADSS was grouped into Groups 1, 2, and 3, reflecting a positive, flat, and negative strain slope at the diastasis phase, respectively (Figure 2).

The *F*-test probability and the pseudo-*R*^2^ were as follows: (1) ΔLVEF Model 1: 0.013 and 0.19; Model 2: <0.01 and 0.29. (2) ΔLGE Model 1: 0.04 and 0.14; Model 2: 0.015 and 0.19.

^a^ ΔLVEF (%) = chronic LVEF − acute LVEF. ΔLGE scar (%) = chronic LGE − acute LGE.

^b^ The covariates in the models were stepwise forward-selected with *p *< 0.3. Model 1: The associations of LADSS with ΔLVEF or ΔLGE scar amount, adjusted for the demographics, heart rate, presence of mitral regurgitation, and LA indices. Model 2: Model 1 + further adjusted for the LV index.

^c^ Statistical significance was set at *p *< 0.017 after Bonferroni correction.

LADSS, left atrial diastasis strain slope; LVEF, left ventricular ejection fraction; LGE, late gadolinium enhancement; BMI, body mass index; LA, left atrium; LAVimax, maximum indexed LA volume; SR, strain rate.

The bold values represent *p* < 0.05, or *p* < 0.017 after Bonferroni correction for LADSS groups, indicating statistical significance.

## Discussion

In this ancillary study of the LAST-PASS MRI sub-study, we particularly focused on a specific feature of the LA strain curve slope at the diastasis phase, LADSS, and identified its clinical significance and meaning in a post-STEMI cohort. Our primary findings are as follows: (1) The LADSS is an important feature of the LA strain curve, with class switch occurring in 55.9% longitudinally in the current post-STEMI cohort. Participants who experienced worsening per the LADSS groups showed poor recovery of LVEF and changes in LGE scar size, especially those who switched from Group 2 in the acute phase to Group 3 in the chronic phase. (2) Cases in LADSS Group 3 manifested suboptimal clinical characteristics compared to their Group 1 counterparts, such as a higher heart rate in the acute phase, and, lower LVEF and weaker LA passive SR in the chronic phase. (3) LADSS was cross-sectionally associated with heart rate, LVEF, and sex in both acute and chronic phases, as well as with LA passive SR in the chronic phase. (4) Acute phase LADSS Groups 2 and 3 were predictors of poor recovery of LVEF when adjusted for the demographics and LA indices; LADSS Group 2 remained a predictor even after adjustment for LV scar amount and acute phase LVEF. Overall, LADSS emerged as a morphological marker of the LA strain curve indicative of the current LV hemodynamics and its recovery in post-anterior STEMI.

LADSS is a unique marker of the diastasis phase, a transitional period between the early LV diastolic (i.e., passive LA emptying) and active LA emptying phases. The diastasis phase has not been clearly described by any indices derived from the LA volumetric, strain, or strain rate (8, 9, 19). The initial point of the diastasis phase is not clear-cut on the LA strain curve due to its transitional nature, but our strategy of utilizing strain rate to differentiate its positive, flat, and negative slope was successful. The physiological mechanism behind the positive, flat, and negative LADSS is explained by the balance between mitral valve (MV) and pulmonary venous (PV) flow volumes (20, 21). In cases with good LV diastolic function, the LV generates enough suction to draw blood from the LA during the early diastolic phase, resulting in low pressure in the LA and allowing room for LA expansion during the diastasis phase. This leads to an increase in LA volume with a larger PV flow volume than MV flow volume, resulting in a positive LADSS, i.e., Group 1. Indeed, this phenomenon is what we have observed on cine images as a small enlargement of the LA chamber during the diastasis phase. Conversely, in cases of LV diastolic dysfunction, the MV E-wave flow shows an elongated deceleration time on echocardiography (20). The LV’s inability to create sufficient suction leads to a relative increase in LA pressure, restricting LA expansion during the diastasis phase. This results in a gradual decrease in LA volume and a negative LADSS (Group 3). LADSS Group 2 serves as a transitional state between Groups 1 and 3, although this group may encompass cases of pseudo-normalization in the advanced stage of LV diastolic dysfunction, considering the pseudo-normalization of MV and PV flows (20). In this context, LADSS may represent a unique pattern of LA–LV interdependency during diastole.

LADSS was not a fixed feature of the strain curve but was rather a timely reflection of the current hemodynamics. The flexibility of LA volumetric curve morphology has been recognized from a cardiac MRI study utilizing dobutamine or glycopyrrolate stress (22). In that study, the positive slope of the LA volume curve during the diastasis phase (which corresponds to the same timeframe as LADSS) flattened during stress in elderly participants, whereas younger participants maintained a positive slope; this phenomenon was proposed as a marker of LV diastolic dysfunction in the elderly. Given the parallel relationship between LA volume and strain curves, this study may support LADSS as a potential marker of LV diastolic dysfunction. A weak LA passive SR correlated with LADSS Group 3 in the chronic phase. It is noted that the LA passive SR is the counterpart of the LV early diastolic strain rate (*E*'sr), an index indicative of LV diastolic dysfunction. This index also finds utility in the evaluation of LV diastolic function, including the *E*/*E*'sr ratio (23) and the strain relaxation index (SRI) (derived from the ratio of isovolumetric relaxation time during the diastolic phase to *E*'sr, obtained through circumferential LV strain measurements using tagged MRI assessment) (24). In this context, LA passive SR could serve as another potential marker of LV diastolic dysfunction. Female sex was associated with acute phase LADSS Group 3 compared to combined Groups 1 and 2 and marginally associated with chronic phase LADSS Group 3 compared to Group 1. This might reflect post-STEMI clinical differences by sex (25) or original differences in the shape of the LA strain curve by sex, although this was not evident in the present study.

In addition to clinically used metrics such as the ratio of the transmitral early filling velocity (*E*) to the late filling velocity (*A*) and to the early relaxation tissue velocity (*e*′) on echocardiography (*E*/*A* and *E*/*e*′, respectively), multiple efforts have been made to assess diastolic dysfunction using both echocardiography (19) and cardiac MRI (26, 27). Few diastolic dysfunction indices, however, have focused on the diastasis phase—the transitional state between the early LV diastolic (i.e., passive LA emptying) and active LA emptying phases. For example, from the LV perspective, the diastolic dysfunction indices explored were the peak filling rate (PFR) (calculated as the maximum differential of the LV volume curve and observed to increase with dobutamine stress cardiac MRI: less pronounced in elderly subjects than in young) (22), time to peak untwisting rate (which is delayed in aortic stenosis patients compared to controls in a study utilizing tagging MRI) (28), and the SRI (as previously mentioned, *E*'sr used in this index is the counterpart of LA passive SR; SRI was predictive of heart failure and atrial fibrillation after adjusting for conventional risk factors in the multi-ethnic study of atherosclerosis) (24). From the LA perspective, the indices explored were LAVimax (increased with worsening diastolic dysfunction according to echocardiography; however, diastolic dysfunction itself was a stronger predictor of mortality than LAVimax) (29), LAVimin [LAVimin ≥ 23 ml/m^2^ by cardiac MRI was a cutoff to detect elevated LV end-diastolic pressure (LVEDP) of ≥12 mmHg, with 86% sensitivity and 63% specificity and a stronger association than LAVimax] (30), LAEF passive (reduced reserve of LAEF passive during dobutamine stress MRI holds prognostic value and is considered a marker of ischemia-induced diastolic dysfunction) (6), and LA reservoir strain (echocardiographic LA reservoir strain cutoffs of 35%, 24%, and 19% differentiate LV diastolic dysfunction grades 0, 1, 2, and 3 in patients with preserved LVEF (5); LA reservoir strain-defined diastolic dysfunction of ≤24% has been associated with incident heart failure in the elderly, independently of LAVi, in a study of asymptomatic elderly subjects with non-ischemic heart failure risk factors (13)). Overall, incorporating LA indices enhances the accuracy of diagnosing, stratifying prognosis, and monitoring treatment of LV diastolic dysfunction and heart failure with preserved EF (HFpEF) (19). In this context, LADSS may offer a distinctive contribution as a unique marker of LV diastolic dysfunction, with a focus on the diastasis phase.

Concurrent observation of the LA volumetric, strain, and strain rate curves offers an efficient approach to comprehending hemodynamics beyond concentrating solely on extracted few metrics. “The Atlas of LA strain,” a compilation of LA volumetric, strain, and strain rate curves collected both in the acute and chronic phases, utilizing consistent *x*-axis (time) and *y*-axis (volume, strain, or strain rate) scales across all cases, has proven successful in this pursuit. Our study question, “What is the clinical meaning of LADSS,” was born from a meticulous visual examination of cases. Interestingly, such an idea of comprehensive observation of the LA strain curve shape is embraced in an AI-based echocardiography LA strain curve shape analysis and prognostication in the general population (31). In that study, the representative LA curve, acting as a centroid of the cluster, presented a positive or flat LADSS (corresponding to our Groups 1 and 2) in those with good prognosis, while the cluster with the worst prognosis presented a negative LADSS curve (corresponding to our Group 3) in the validation cohort (31). This aligns with the current observation that LADSS Group 3 presented suboptimal clinical characteristics. Notably, the weak LA passive SR was a feature of the cluster with the most unfavorable prognosis in the AI-based LA strain curve shape analysis study (31). This possibly aligns with our present result linking weak LA passive SR to LADSS Group 3 in the chronic phase.

While we hypothesized that LV diastolic dysfunction may be an underlying mechanism of LADSS, we could not directly verify it due to the study design. A validation study utilizing echocardiography is warranted, in which we also anticipate gaining insights into the potential pseudo-normalization in LADSS.

## Conclusion

In conclusion, LADSS serves as a marker of instantaneous LV hemodynamics, and its temporal evolution in post-anterior STEMI is independent of LA or LV indices. LADSS reflects a unique pattern of LA–LV interdependence during diastole. Further investigation of LADSS in other etiologies including heart failure associated with diastolic dysfunction is warranted, as well as its value as a marker in prognostication.

## Data Availability

The raw data supporting the conclusions of this article will be made available by the authors without undue reservation.
