# Supplementary material for: Left atrial diastasis strain slope is a marker of hemodynamic recovery in post-ST elevation myocardial infarction: the Laser Atherectomy for STemi, Pci Analysis with Scintigraphy Study (LAST-PASS)
Source: Front Radiol. 2024 Feb 21;4:1294398. doi: 10.3389/fradi.2024.1294398 (PMC10914933; doi:10.3389/fradi.2024.1294398)
Supplement: Supplementary file 1 [file Datasheet1.docx]

**Supplemental Material S1. MRI protocol.**
A standard cardiac MRI protocol including cine and LGE was performed using 1.5 or 3.0 Tesla magnets at the 5 LAST-PASS MRI facilities. The MRI scanners at each facility were as follows: Site #01 (Tokai University), 1.5T Ingenia and 1.5T dStream, Philips Healthcare, Best, the Netherlands; Site #02 (Ogaki municipal hospital), 1.5T Achieva, Philips Healthcare, Best, the Netherlands; Site #03 (Ageo central hospital), 1.5T Optima MR360 advance, General Electric, Milwaukee, WI; Site #06 (Osaka general hospital), 1.5T Achieva, Philips Healthcare, Best, the Netherlands; and Site #17 (Tottori University), 3T Skyra, Siemens Healthineers, Erlangen, Germany.

All MRI images were acquired under EKG gating at end-expiration. Participants eligible for contrast received intravenous administration of 0.15 mmol/kg gadobutrol (Gadovist, Bayer, Berlin, Germany). Single 2- and 4-chamber long-axes and multi-slice short-axis cine images with whole-heart coverage were acquired. A 2D-steady-state free precession (SSFP) sequence was utilized with the following typical imaging parameters: flip angle = 50°; TR/TE = 2.8/1.4 ms; pixel bandwidth 1033 Hz/pixel; matrix = 192 × 200; voxel size = 1.5 × 1.5 × 8 mm; slice thickness = 8mm; slice gap = 2mm; 30 phases per cardiac cycle. For the 3.0T scanner, the typical parameters for cine images were as follows: flip angle = 51°; TR/TE = 39.1/1.4 ms; pixel bandwidth 960 Hz/pixel; matrix = 208 × 149; voxel size = 0.8 × 0.8 × 8 mm; slice thickness = 8mm; slice gap = 2mm; 30 phases per cardiac cycle. Multi-slice LGE images covering the entire LV were acquired at 17±3 min after contrast administration using a standard 2D-inversion recovery gradient echo sequence or a segmented phase-sensitive inversion recovery (PSIR) gradient echo sequence. The inversion time (TI) was adjusted in each scan to null the signal of the remote myocardium. The following were the typical imaging parameters on a 1.5T scanner: flip angle = 25°; TR/TE = 6.2/3.0ms; pixel bandwidth 233Hz/pixel; matrix = 208 × 217; voxel size = 1.2 × 1.2 × 8 mm; slice thickness = 8mm; slice gap = 2mm, TI time = 265ms. For the 3.0T scanner, the typical parameters for LGE images were as follows: flip angle = 20°; TR/TE = 758.0/1.5ms; pixel bandwidth 465Hz/pixel; matrix = 256 × 124; voxel size = 1.4 × 1.4 × 8 mm; slice thickness = 8mm; slice gap = 2mm, TI time = 360ms.
