# Supplementary material for: Left atrial diastasis strain slope is a marker of hemodynamic recovery in post-ST elevation myocardial infarction: the Laser Atherectomy for STemi, Pci Analysis with Scintigraphy Study (LAST-PASS)
Source: Front Radiol. 2024 Feb 21;4:1294398. doi: 10.3389/fradi.2024.1294398 (PMC10914933; doi:10.3389/fradi.2024.1294398)
Supplement: Supplementary file 2 [file Datasheet2.doc]

# Supplemental Material S2. Reproducibility of LA strain analysis by MTT software.

The reproducibility of left atrial (LA) strain analysis was investigated using 23 randomly selected cases. A single observer performed the LA strain analysis twice, 14 days apart, using the multimodality tissue tracking software (MTT; version 6.1.4826, Toshiba, Japan) (1). The contour was initially drawn at the largest LA volume and then propagated throughout the entire cardiac phases, with the software automatically tracking the LA contours. Intra-reader reproducibility was assessed using the Bland-Altman plot and interclass correlation coefficients (ICC). The smallest detectable change (SDC) and coefficient of variation (%CV) were calculated. Participant characteristics included 82.6% males, a mean age of 64.5±11.4 year old, and a BMI of 23.4±2.9 (kg/m2). Overall, the ICC was excellent, with values higher than 0.9 for all LA analysis indices. The SDC was sufficiently low to detect differences between cases. The %CV was less than 10% except for the LAEF passive (Table). Scatterplot and Bland-Altman plot graphs for each LA index were presented in the Figure.

**References**

1. Zareian M, Ciuffo L, Habibi M, Opdahl A, Chamera EH, Wu CO, Bluemke DA, Lima JAC, Venkatesh BA. Left atrial structure and functional quantitation using cardiovascular magnetic resonance and multimodality tissue tracking: Validation and reproducibility assessment. J Cardiovasc Magn Reson (2015) 17:1–13. doi: 10.1186/s12968-015-0152-y

**Table. Intra-observer reproducibility of LA strain analysis.**

| **LA indices** | **unit** | **First analysis** | **Second analysis** | ***P* value** | **Mean±LoA** | **ICC** | ***P* value** | **SDC** | **%CV** |
| --- | --- | --- | --- | --- | --- | --- | --- | --- | --- |
| LAVmax | ml | 61.4±21.6 | 59.0±22.5 | <0.01** | 2.4±7.4 | 0.98 | <0.01** | 1.48 | 4.85 |
| LAVmin | ml | 31.2±13.6 | 29.8±14.5 | 0.042* | 1.4±5.9 | 0.97 | <0.01** | 1.46 | 7.78 |
| LAVpreA | ml | 49.3±17.4 | 46.5±17.7 | <0.01** | 2.7±8.2 | 0.96 | <0.01** | 2.32 | 6.45 |
| LAEFtotal | % | 49.9±9.6 | 50.8±10.9 | 0.13 | -0.96±5.7 | 0.96 | <0.01** | 1.62 | 3.22 |
| LAEFpassive | % | 19.1±9.0 | 20.6±8.0 | 0.024* | -1.5±5.8 | 0.93 | <0.01** | 2.18 | 14.26 |
| LAEFbooster | % | 38.1±8.6 | 38.2±11.2 | 0.94 | -0.05±7.0 | 0.94 | <0.01** | 2.43 | 5.80 |
| LA reservoir strain | % | 26.6±8.4 | 27.3±7.8 | 0.24 | -0.64±5.0 | 0.95 | <0.01** | 1.58 | 5.19 |
| LA booster pump strain | % | 15.5±6.5 | 15.7±5.8 | 0.52 | -0.28±3.9 | 0.95 | <0.01** | 1.22 | 7.29 |
| LA reservoir SR | sec-1 | 1.1 (0.97 - 1.5) | 1.3±0.43 | 0.99 | 0.01±0.41 | 0.90 | <0.01** | 0.18 | 8.11 |
| LA passive SR | sec-1 | -0.82 (-1.02 - -0.56) | -0.94±0.50 | 0.99 | 0.01±0.19 | 0.98 | <0.01** | 0.04 | -7.45 |
| LA booster pump SR | sec-1 | -1.5±0.65 | -1.6±0.69 | 0.14 | 0.06±0.35 | 0.96 | <0.01** | 0.10 | -7.04 |

LA, left atrium; LoA, limits of agreement; ICC, interclass correlation coefficient; SDC, smallest detectable change; %CV, coefficient of variation; LAVmax, LA maximum volume; LAVpreA, LA pre-atrial kick volume; LAVmin, LA minimum volume; LAEF, LA ejection fraction; SR, strain rate.

** *P*<0.01; **P*<0.05.

**Figure. Intra-observer reproducibility of LA strain analysis.**

**
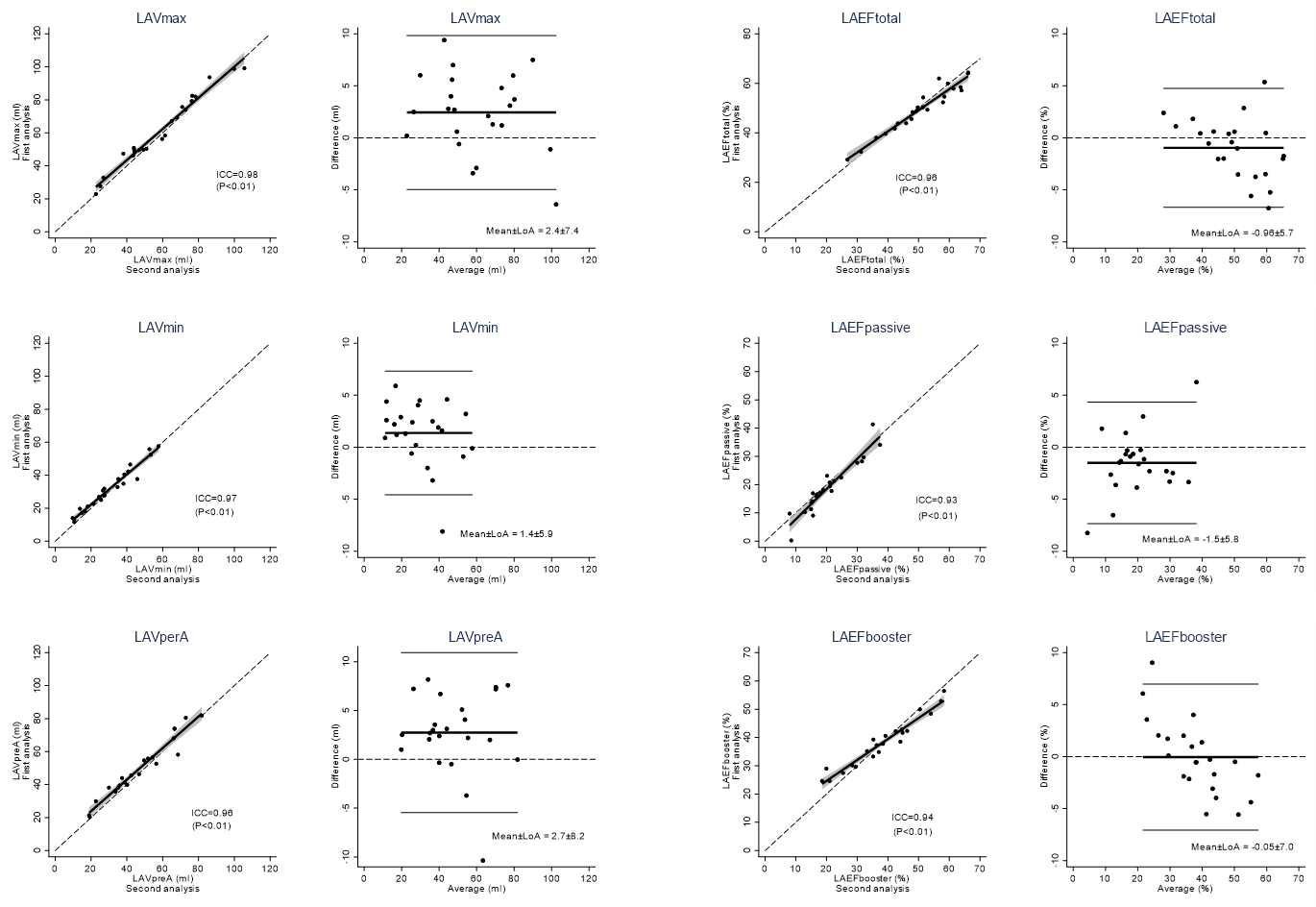
**

**
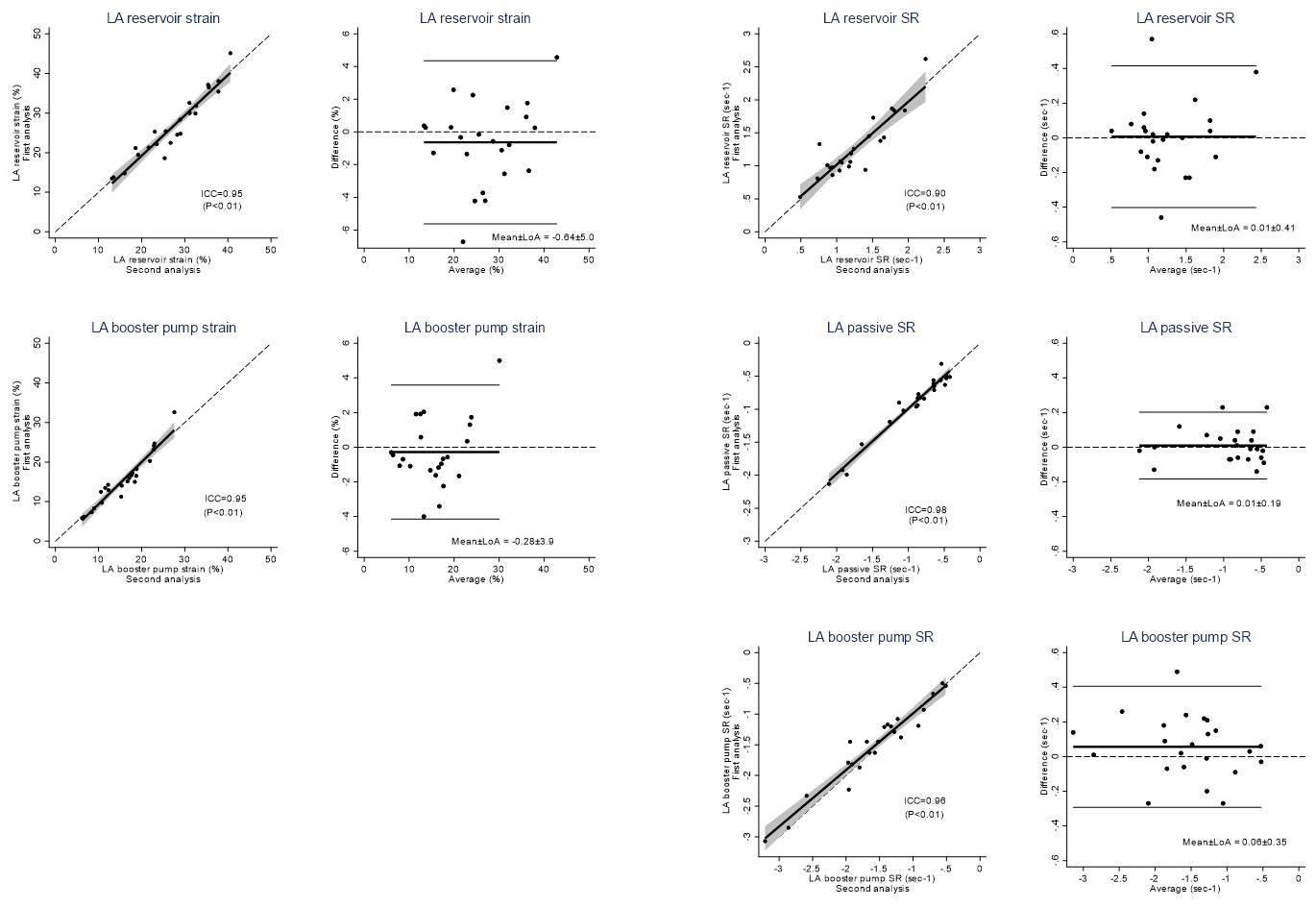
**

Scatterplot and Bland-Altman plot graphs were presented for each LA index. A consistent x- and y-axes range was employed for the volume, EF, strain, and SR indices within the same category, except for LA reservoir SR.

LA, left atrium; ICC, interclass correlation coefficient; LoA, limits of agreement; LAVmax, LA maximum volume; LAVpreA, LA pre-atrial kick volume; LAVmin, LA minimum volume; LAEF, LA ejection fraction; SR, strain rate.
