# Supplementary material for: Left atrial diastasis strain slope is a marker of hemodynamic recovery in post-ST elevation myocardial infarction: the Laser Atherectomy for STemi, Pci Analysis with Scintigraphy Study (LAST-PASS)
Source: Front Radiol. 2024 Feb 21;4:1294398. doi: 10.3389/fradi.2024.1294398 (PMC10914933; doi:10.3389/fradi.2024.1294398)
Supplement: Supplementary file 3 [file Datasheet3.doc]

# Supplemental Material S3. LADSS group identification protocol.

LADSS was grouped into 1, 2, and 3, reflecting a positive, flat, and negative slope, respectively (**Figure 2**). A systematic method to determine the LADSS group was developed from the receiver operating characteristic curve (ROC curve) analysis that utilized the number of phases that exhibit positive strain rate values during the diastasis phase against visual assessment classification, considering strain rate is a differential of strain curve. First, all the LA strain curves were assigned to the LADSS groups by visual assessment. Then, the number of phases that presented positive strain rate values during the diastasis phase, specifically between the two negative peaks in the atrial systolic phase (i.e., ventricular diastolic phase), were counted in the individual case. The number of phases that best separated the visual LADSS group 1 (positive slope) from the remaining groups (i.e., that presented the highest positive predictive value (PPV) were identified from the ROC curve analysis. Similarly, the number of phases that best separated the visual LADSS group 3 (negative slope) from the remaining groups were identified from the ROC curve. Overall, when the number of positive strain rate phases was 3 and more, it best matched the visual LADSS group 1 (positive slope) with sensitivity (Se) = 52.6%, specificity (Sp) = 97.7%, PPV = 90.9%, negative predictive value (NPV) = 82.5%, and accuracy = 84.0%. Concurrently, when the number of positive strain rate phases was 0, it best matched the visual LADSS group 3 (negative slope) with Se = 71.8%, Sp = 97.9%, PPV = 98.3%, NPV = 67.7%, and accuracy = 81.6%. Therefore, the final protocol to identify LADSS groups 1, 2, and 3 was defined as the number of positive strain rate phases during the diastasis phase being 3 and more, 1 or 2, and 0, respectively.
