# Supplementary material for: Left atrial diastasis strain slope is a marker of hemodynamic recovery in post-ST elevation myocardial infarction: the Laser Atherectomy for STemi, Pci Analysis with Scintigraphy Study (LAST-PASS)
Source: Front Radiol. 2024 Feb 21;4:1294398. doi: 10.3389/fradi.2024.1294398 (PMC10914933; doi:10.3389/fradi.2024.1294398)
Supplement: Supplementary file 4 [file Datasheet4.doc]

# Supplemental Material S4. Atlas of LA strain in STEMI cohort.

The left atrial (LA) volume, strain, and strain rate in the acute and chronic phases were graphically summarized in all cases by the LA diastasis strain slope (LADSS) group to provide an overview of the trends in LA strain curve shapes within each LADSS group. LADSS was categorized as 1, 2, or 3, reflecting a positive, flat, or negative strain slope at the diastasis phase, respectively (as shown in the figure below, copied from Figure 2, panel A).

**Figure. LADSS group**


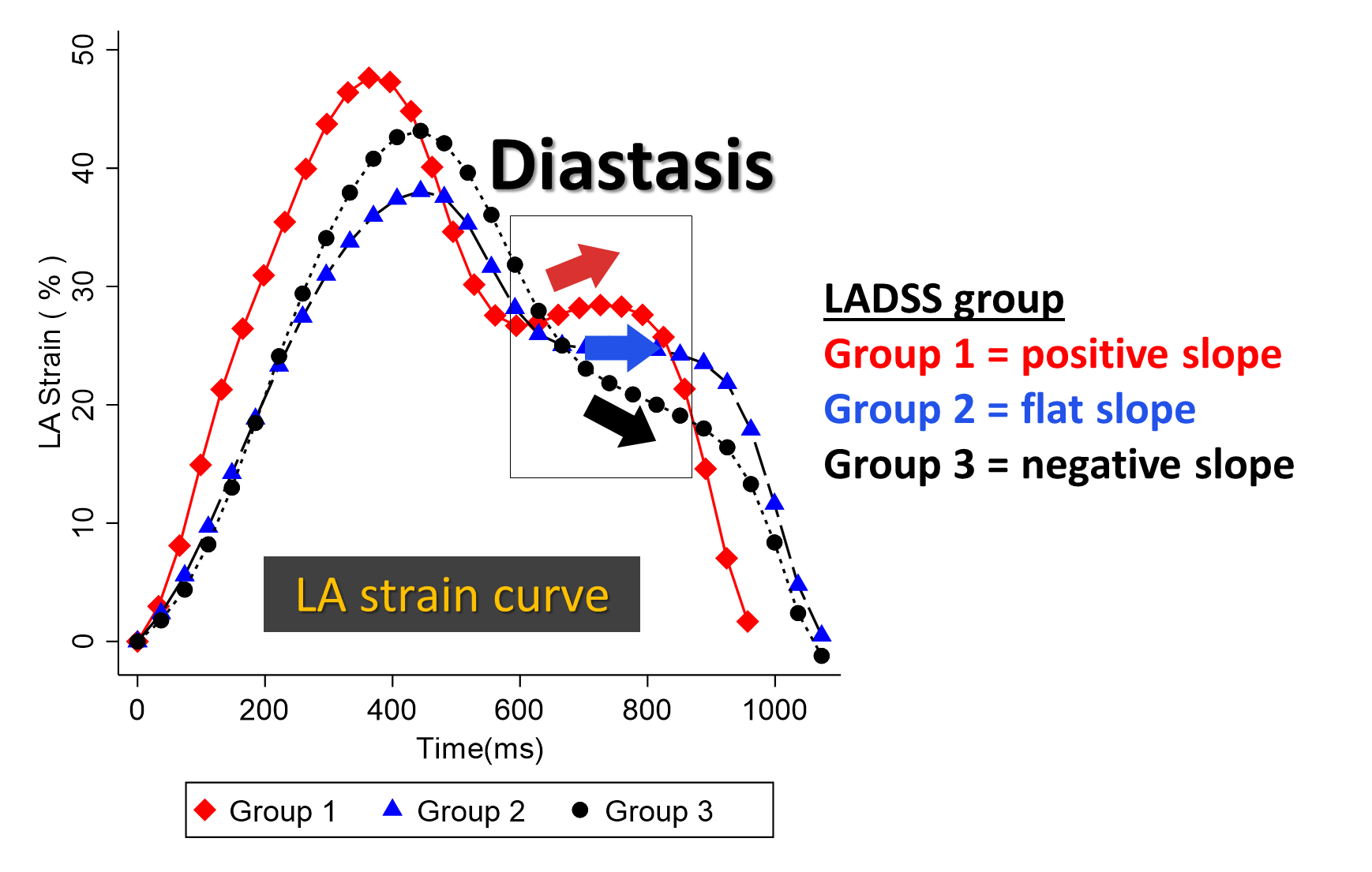


LADSS group identification protocol was summarized in **Supplemental Material S3**. LADSS, left atrial diastasis strain slope.

On Page 3, left ventricular ejection fraction (LVEF) and LV scar size by late gadolinium enhancement (LGE) for the LADSS group combinations in both the acute and chronic phases are presented in a table. The LV function and scar size generally improved from acute to chronic phases when the LADSS category switched to Group 1, or, when the LADSS was maintained longitudinally.

From page 4, the acute phase LA analysis graphs are displayed on the left side, while the chronic phase graphs are shown on the right side. In each phase, the following curves were presented from left to right in this order: the LA volumetric curve, the LA strain curve, the LA strain rate curve, and the superimposed LA strain and strain rate curves. It is noted that consistent x-axis (time) and y-axis (volume, strain, or strain rate) scales were applied throughout all cases, except for the rightmost superimposed LA strain and strain rate curves.

| Page | Section # | Acute phase LADSS group | Chronic phase LADSS group |
| --- | --- | --- | --- |
| 4 | 1. | Group 1 | Group 1 |
| 5 | 2. | Group 2 | Group 1 |
| 6 | 3. | Group 3 | Group 1 |
| 8 | 4. | Group 1 | Group 2 |
| 8 | 5. | Group 2 | Group 2 |
| 9 | 6. | Group 3 | Group 2 |
| 10 | 7. | Group 1 | Group 3 |
| 11 | 8. | Group 2 | Group 3 |
| 13 | 9. | Group 3 | Group 3 |
| 16 | 10. | Group 1 | N/A |
| 16 | 11. | Group 2 | N/A |
| 17 | 12. | Group 3 | N/A |

# Table. LVEF (%) and LGE scar amount (%) by the LADSS group combinations in the acute and chronic phases.

LVEF and LGE scar amount in both the acute and chronic phases, along with the number of cases in each category, are summarized based on the LADSS Group combinations. LV function and scar size generally improved from the acute to chronic phases when the LADSS category switched to Group 1, or, when the LADSS was maintained longitudinally.

LVEF, left ventricular ejection fraction; LGE, late gadolinium enhancement; LA, left atrium; LADSS, LA diastasis strain slope.

# 1. LADSS Group 1 🡪 Group 1

| **Acute phase** | **Chronic phase** |
| --- | --- |

# 2. LADSS Group 2🡪 Group 1

| **Acute phase** | **Chronic phase** |
| --- | --- |

# 3. LADSS Group 3 🡪 Group 1

| **Acute phase** | **Chronic phase** |
| --- | --- |

# 4. LADSS Group 1 🡪 Group 2

| **Acute phase** | **Chronic phase** |
| --- | --- |

# 5. LADSS Group 2 🡪 Group 2

| **Acute phase** | **Chronic phase** |
| --- | --- |

# 6. LADSS Group 3 🡪 Group 2

| **Acute phase** | **Chronic phase** |
| --- | --- |

# 7. LADSS Group 1 🡪 Group 3

| **Acute phase** | **Chronic phase** |
| --- | --- |

# 8. LADSS Group 2 🡪 Group 3

| **Acute phase** | **Chronic phase** |
| --- | --- |

# 9. LADSS Group 3 🡪 Group 3

| **Acute phase** | **Chronic phase** |
| --- | --- |

# 10. LADSS Group 1 🡪 N/A

| **Acute phase** | **Chronic phase** |
| --- | --- |

# 11. LADSS Group 2 🡪 N/A

| **Acute phase** | **Chronic phase** |
| --- | --- |

# 12. LADSS Group 3 🡪 N/A

| **Acute phase** | **Chronic phase** |
| --- | --- |
