# Supplementary material for: Left atrial diastasis strain slope is a marker of hemodynamic recovery in post-ST elevation myocardial infarction: the Laser Atherectomy for STemi, Pci Analysis with Scintigraphy Study (LAST-PASS)
Source: Front Radiol. 2024 Feb 21;4:1294398. doi: 10.3389/fradi.2024.1294398 (PMC10914933; doi:10.3389/fradi.2024.1294398)
Supplement: Supplementary file 5 [file Datasheet5.doc]

# Supplemental Material Table S5. Participant characteristics and cardiac MRI indices by LADSS groups.

|  | **Acute phase, by LADSS** | | | ***P* value** | **Chronic phase, by LADSS** | | | ***P* value** |
| --- | --- | --- | --- | --- | --- | --- | --- | --- |
|  | **Group 1** | **Group 2** | **Group 3** |  | **Group 1** | **Group 2** | **Group 3** |  |
| **Participant characteristics** | | | | | | | | |
| N (%) | 16 (24.2) | 19 (28.8) | 31 (47.0) | N/A | 20 (33.9) | 13 (22.0) | 26 (44.1) | N/A |
| Age at MRI (y/o) | 64.1±11.6 | 59.8±9.7 | 64.7±12.9 | 0.35 | 61.0±12.0 | 62.5±10.4 | 66.0±11.8 | 0.33 |
| Male, N (%) | 15 (93.8) | 18 (94.7) | 24 (77.4) | 0.14 | 19 (95.0) | 11 (84.6) | 21 (80.8) | 0.37 |
| BMI at MRI (kg/m2) | 22.6±1.9 | 23.3  (21.6 - 24.1) | 23.1±3.1 | 0.41 | 23.2±1.9 | 23.7±3.7 | 22.1  (20.6 - 24.4) | 0.50 |
| Heart rate at MRI (bpm) | 61.9±8.7* | 70.6±12.0 | 73.5±11.9* | **<0.01** | 57.7±4.9 | 59.5±9.9 | 62.2±10.0 | 0.40 |
| Max_CKMB (IU/L) | 318.7±164.8 | 428±261.1 | 353  (194 - 584) | 0.54 | 352.0±256.6 | 314 (222 - 584) | 395.5 (274 - 540) | 0.46 |
| TIMI flow before index PCI |  |  |  | 0.55 |  |  |  | 0.33 |
| TIMI 0 | 12 (75.0) | 16 (88.9) | 27 (87.1) |  | 15 (75.0) | 10 (83.3) | 24 (92.3) |  |
| TIMI 1 | 4 (25.0) | 2 (11.1) | 4 (12.9) |  | 5 (25.0) | 2 (16.7) | 2 (7.7) |  |
| TIMI flow post index PCI |  |  |  | 0.78 |  |  |  | 0.81 |
| TIMI 0 | 0 (0) | 1 (5.3) | 0 (0) |  | 1 (5.0) | 0 (0) | 0 (0) |  |
| TIMI 1 | 0 (0) | 0 (0) | 0 (0) |  | 0 (0) | 0 (0) | 0 (0) |  |
| TIMI 2 | 0 (0) | 0 (0) | 1 (3.2) |  | 0 (0) | 0 (0) | 1 (3.9) |  |
| TIMI 3 | 16 (100) | 18 (94.7) | 30 (96.8) |  | 19 (95.0) | 13 (100) | 25 (96.2) |  |
| BNP at 6 months post-STEMI (pg/ml) | 55.7  (15.9 - 63) | 49.6  (22.6 - 122.5) | 84.9  (33 - 177.1) | 0.17 | 47.2  (25.4 - 63.0) | 38.4  (16.5 - 87.0) | 86.9  (48.0 - 178.0) | 0.15 |
| Smoking status at hospitalization |  |  |  | 0.88 |  |  |  | 0.96 |
| Never | 5 (31.3) | 4 (21.1) | 10 (32.3) |  | 6 (30.0) | 3 (23.1) | 7 (26.9) |  |
| Current smoker | 7 (43.8) | 10 (52.6) | 14 (45.2) |  | 9 (45.0) | 7 (53.9) | 12 (46.2) |  |
| Past smoker | 4 (25.0) | 4 (21.1) | 7 (22.6) |  | 4 (20.0) | 3 (23.1) | 7 (26.9) |  |
| Unknown | 0 (0) | 1 (5.3) | 0 (0) |  | 1 (5.0) | 0 (0) | 0 (0) |  |
| Diabetes mellitus | 4 (25.0) | 1 (5.3) | 8 (25.8) | 0.17 | 3 (15.0) | 3 (23.1) | 5 (19.2) | 0.91 |
| Dyslipidemia | 11 (68.8) | 13 (68.4) | 20 (64.5) | 0.94 | 14 (70.0) | 8 (61.5) | 18 (69.2) | 0.86 |
| Hypertension | 11 (68.8) | 8 (42.1) | 19 (61.3) | 0.24 | 12 (60.0) | 5 (38.5) | 16 (61.5) | 0.35 |
| Mitral regurgitation present, n (%) | 3 (18.8) | 5 (26.3) | 12 (38.7) | 0.33 | 4 (20.0) | 2 (15.4) | 6 (23.1) | 0.92 |
| Pericardial effusion present, n (%) | 5 (31.3) | 11 (57.9) | 16 (51.6) | 0.26 | 1 (5.0) | 0 (0) | 1 (3.9) | 1.0 |
| Pleural effusion present, n (%) | 4 (25.0) | 3 (15.8) | 9 (29.0) | 0.57 | 0 (0) | 1 (7.7) | 1 (3.9) | 0.70 |
| **Cardiac MRI indices** | | | | | | | | |
| **LV indices** |  |  |  |  |  |  |  |  |
| LVEDVi (ml/m2) | 95.1±16.1 | 91.0±11.6 | 98.6±22.8 | 0.38 | 93.5±21.0 | 82.6  (73.2 - 120.4) | 98.4  (82.0 - 108.3) | 0.73 |
| LVESVi (ml/m2) | 56.9±14.2 | 53.6±8.7 | 64.7±22.9 | 0.22 | 46.7  (36.3 - 58.5) | 42.4  (40.6 - 64.2) | 52.2  (45.3 - 69.3) | 0.32 |
| LVMi (g/m2) | 63.0  (58.6 - 68.4) | 70.8±11.3 | 72.3±12.5 | 0.080 | 53.5±6.6 | 57.0±11.0 | 58.9±10.0 | 0.15 |
| LVEF (%) | 40.7±6.2 | 41.1±6.3 | 35.8±10.0 | 0.13 | 48.7±8.6* | 44.8±9.4 | 41.8±9.9* | **0.053** |
| LGE scar size (%) | 17.9±8.9 | 21.9±11.5 | 23.5±14.9 | 0.43 | 13.2±10.2 | 11.1  (9.0 - 16.2) | 19.5±11.3 | 0.14 |
| **LA indices** |  |  |  |  |  |  |  |  |
| LAVimax (ml/m2) | 31.5±8.3 | 30.3±6.5 | 34.0±10.3 | 0.32 | 31.4±13.2 | 30.8±9.8 | 34.7±11.2 | 0.52 |
| LAVipreA (ml/m2) | 24.9±7.4 | 24.2±7.0 | 27.3±9.2 | 0.39 | 21.3  (14.2 - 30.5) | 22.4  (20.9 - 26.2) | 27.5±8.9 | 0.43 |
| LAVimin (ml/m2) | 15.8±6.0 | 13.5  (11.1 - 16.1) | 16.4  (10.9 - 23.5) | 0.41 | 10.6  (7.7 - 19.9) | 12.4  (10.5 - 16.2) | 17.6±8.5 | 0.39 |
| LAEF total (%) | 51.1±9.5 | 54.6  (50.2 - 58.8) | 52.7  (44.1 - 59.6) | 0.84 | 56.9±11.8 | 54.7±9.4 | 50.8±11.7 | 0.19 |
| LAEF passive (%) | 21.2±6.7 | 22.6  (16.7 - 24.9) | 20.1±7.7 | 0.85 | 24.1±7.5 | 22.4±6.4 | 20.1±8.2 | 0.21 |
| LAEF booster pump (%) | 38.2±9.4 | 41.2  (37.1 - 44.6) | 37.8±11.8 | 0.83 | 46.2  (36.5 - 49.5) | 41.9±9.4 | 38.4±12.7 | 0.30 |
| LA reservoir strain (%) | 26.5±4.7 | 27.1±7.3 | 25.6±10.1 | 0.68 | 30.3±11.0 | 29.5±7.8 | 28.1±9.9 | 0.73 |
| LA booster pump strain (%) | 15.9±5.3 | 14.9±5.5 | 13.5±6.1 | 0.38 | 16.2±7.4 | 15.8±5.2 | 14.9±6.5 | 0.81 |
| LA reservoir SR (sec^-1^) | 1.1  (1.0 - 1.3) | 1.2±0.3 | 1.2±0.4 | 0.59 | 1.3±0.4 | 1.2±0.3 | 1.2±0.5 | 0.65 |
| LA passive SR (sec^-1^) | -0.9  (-1.0 - -0.8) | -1.1±0.4 | -0.8  (-1.1 - -0.5) | 0.35 | -1.1±0.4* | -1.1±0.4 | -0.7  (-1.2 - -0.6)* | **0.038** |
| LA booster pump SR (sec^-1^) | -1.4±0.5 | -1.5±0.6 | -1.5±0.6 | 0.74 | -1.5±0.6 | -1.4±0.5 | -1.4±0.6 | 0.80 |
| LADSS in the acute phase (N, [%]) |  |  |  | N/A |  |  |  | 0.65 |
| Group 1 | 16 (100) | 0 (0) | 0 (0) |  | 6 (30.0) | 3 (23.1) | 4 (15.4) |  |
| Group 2 | 0 (0) | 19 (100) | 0 (0) |  | 6 (30.0) | 5 (38.5) | 7 (26.9) |  |
| Group 3 | 0 (0) | 0 (0) | 31 (100) |  | 8(40.0) | 5 (38.5) | 15 (57.7) |  |
| LADSS in the chronic phase (N, [%]) |  |  |  | 0.68 |  |  |  | N/A |
| Group 1 | 6 (37.5) | 6 (31.6) | 8 (25.8) |  | 20 (100) | 0 (0) | 0 (0) |  |
| Group 2 | 3 (18.8) | 5 (26.3) | 5 (16.1) |  | 0 (0) | 13 (100) | 0 (0) |  |
| Group 3 | 4 (25.0) | 7 (36.8) | 15 (48.4) |  | 0 (0) | 0 (0) | 26 (100) |  |
| Chronic phase MRI not scanned | 3 (18.8) | 1 (5.3) | 3 (9.7) |  | - | - | - |  |

LADSS was grouped into 1, 2, and 3, reflecting a positive, flat, and negative strain slope at the diastasis phase, respectively (**Figure 2**). *Pairs that presented significant differences between Group 1 vs. Group 3 (HR: *p*=0.004; LVEF: *p*=0.041; LA passive SR: *p*=0.037; all by Tukey's HSD test).

MRI, magnetic resonance imaging; LADSS, left atrial diastasis strain slope; BMI, body mass index; CK-MB, creatinine kinese-MB; TIMI, thrombolysis in myocardial infarction; PCI, percutaneous coronary intervention; BNP, brain natriuretic peptide; STEMI, ST-elevation myocardial infarction; LV, left ventricle; LVEDVi, LV end-diastolic volume index; LVESVi, LV end-systolic volume index; LVMi, LV mass index; LVEF, LV ejection fraction; LGE, late gadolinium enhancement; LA, left atrium; LAVimax, LA maximum volume index; LAVipreA, LA pre-atrial kick volume index; LAVimin, LA minimum volume index; LAEF, LA ejection fraction; SR, strain rate; HSD, honestly significant difference.

The bold values represent p<0.05, showing statistical significance.
