# Supplementary material for: Left atrial diastasis strain slope is a marker of hemodynamic recovery in post-ST elevation myocardial infarction: the Laser Atherectomy for STemi, Pci Analysis with Scintigraphy Study (LAST-PASS)
Source: Front Radiol. 2024 Feb 21;4:1294398. doi: 10.3389/fradi.2024.1294398 (PMC10914933; doi:10.3389/fradi.2024.1294398)
Supplement: Supplementary file 6 [file Datasheet6.doc]

# Supplemental Material Table S6. Cross-sectional correlates of LADSS Group 3.

| **Acute phase** | | | | | | |
| --- | --- | --- | --- | --- | --- | --- |
|  | **Univariable** | | **Model 1**^†^ | | **Model 2**^†^ | |
|  | **OR** | ***p* value** | **OR** | ***p* value** | **OR** | ***p* value** |
| Age (y/o) | 1.0 | 0.32 | - |  | - |  |
| Sex (Male) | 0.21 | 0.063 | 0.20 | 0.073 | 0.13 | **0.034** |
| BMI (kg/m2) | 0.98 | 0.78 | - |  | - |  |
| Heart rate (bpm) | **1.1** | **0.025** | 1.1 | **0.027** | 1.04 | 0.20 |
| Mitral regurgitation | 2.1 | 0.17 | - |  | - |  |
| LAVimax (ml/m2) | 1.04 | 0.15 | - |  | - |  |
| LA passive SR (sec^-1^) | 2.0 | 0.23 | 2.8 | 0.092 | - |  |
| LA booster pump SR (sec^-1^) | 0.90 | 0.81 | - |  | - |  |
| LVEF (%) | **0.92** | **0.019** | N/A |  | 0.91 | **0.038** |
| LGE scar amount (%) | 1.0 | 0.29 | N/A |  | - |  |
| **Chronic phase** | | | | | | |
|  | **Univariable** | | **Model 1**^†^ | | **Model 2**^†^ | |
|  | **OR** | ***p* value** | **OR** | ***p* value** | **OR** | ***p* value** |
| Age (y/o) | 1.03 | 0.15 | - |  | 1.1 | 0.14 |
| Sex (Male) | 0.42 | 0.27 | - |  | 0.22 | 0.12 |
| BMI (kg/m2) | 0.94 | 0.51 | - |  | - |  |
| Heart rate (bpm) | 1.1 | 0.10 | 1.1 | 0.076 | 1.1 | **0.040** |
| Mitral regurgitation | 1.4 | 0.64 | - |  | - |  |
| LAVimax (ml/m2) | 1.03 | 0.25 | - |  | - |  |
| LA passive SR (sec^-1^) | **6.1** | **0.018** | 6.7 | **0.013** | - |  |
| LA booster pump SR (sec^-1^) | 1.2 | 0.73 | - |  | - |  |
| LVEF (%) | **0.94** | **0.039** | N/A |  | 0.88 | **<0.01** |
| LGE scar amount (%) | 1.1 | 0.058 | N/A |  | - |  |

LADSS was grouped into 1, 2, and 3, reflecting a positive, flat, and negative strain slope at the diastasis phase, respectively (**Figure 2**).

In the acute phase, the chi-square probability and pseudo R2 of the multivariable Model 1 were < 0.01 and 0.13, and, for Model 2 were <0.01 and 0.18, respectively. Those in the chronic phase Model 1 were <0.01 and 0.12, and, <0.01 and 0.23 for Model 2.

^†^The covariates in the models were stepwise forward selected with p<0.3. Model 1: The determinants of LADSS group 3 from the demographics, heart rate, presence of mitral regurgitation, and LA indices. Model 2: The determinants of LADSS group 3 from Model 1 + LV indices.

LADSS, left atrial diastasis strain slope; BMI, body mass index; LVEF, left ventricular ejection fraction; LGE, late gadolinium enhancement; LA, left atrium; LAVimax, maximum indexed LA volume; SR, strain rate.

The bold values represent p<0.05, showing statistical significance.
