# Supplementary material for: Left atrial diastasis strain slope is a marker of hemodynamic recovery in post-ST elevation myocardial infarction: the Laser Atherectomy for STemi, Pci Analysis with Scintigraphy Study (LAST-PASS)
Source: Front Radiol. 2024 Feb 21;4:1294398. doi: 10.3389/fradi.2024.1294398 (PMC10914933; doi:10.3389/fradi.2024.1294398)
Supplement: Supplementary file 7 [file Datasheet7.doc]

# Supplemental Material Table S7. Associations of the acute phase LADSS with the recovery of LVEF and LGE scar amount in an extended model.

| **Δ LVEF (%)**^†^ | **Model 3**^‡^ | |
| --- | --- | --- |
|  | **β** | **p value** |
| Age (y/o) | - | - |
| Sex (Male) | - | - |
| BMI (kg/m2) | 0.75 | **0.022** |
| Heart rate (bpm) | - | - |
| Mitral regurgitation | -4.1 | **0.030** |
| LAVimax (ml/m2) | 0.27 | **0.012** |
| LA passive SR (sec^-1^) | 3.9 | 0.055 |
| LA booster pump SR (sec-1) | -3.0 | 0.087 |
| LADSS^§^ |  |  |
| Group 1 | reference |  |
| Group 2 | -5.8 | **0.013**^§^ |
| Group 3 | -3.6 | 0.096 |
| Acute phase LVEF (%) | -0.22 | 0.073 |
| Acute phase LGE scar (%) | -0.17 | **0.036** |
| Interactions (LADSS Group × acute phase LVEF) |  |  |
| Group 1 × acute phase LVEF | reference |  |
| Group 2 × acute phase LVEF | - | - |
| Group 3 × acute phase LVEF | - | - |
| **Δ LGE scar (%)**^†^ | **Model 3**^‡^ | |
|  | **β** | **p value** |
| Age (y/o) | - | - |
| Sex (Male) | - | - |
| BMI (kg/m2) | -0.41 | 0.15 |
| Heart rate (bpm) | - | - |
| Mitral regurgitation | 2.5 | 0.14 |
| LAVimax (ml/m2) | - | - |
| LA passive SR (sec^-1^) | - | - |
| LA booster pump SR (sec-1) | - | - |
| LADSS^§^ |  |  |
| Group 1 | reference |  |
| Group 2 | -0.96 | 0.77 |
| Group 3 | -0.14 | 0.95 |
| LVEF (%) | -0.13 | 0.19 |
| LGE scar (%) | -0.41 | **<0.01** |
| Interactions (LADSS Group × acute phase LGE scar) |  |  |
| Group 1 × acute phase LGE scar | reference |  |
| Group 2 × acute phase LGE scar | 0.24 | 0.063 |
| Group 3 × acute phase LGE scar | - | - |

LADSS was grouped into 1, 2, and 3, reflecting a positive, flat, and negative strain slope at the diastasis phase, respectively (**Figure 2**).

The F-test probability and the pseudo R2 were as follows: (1) ΔLVEF Model 3: <0.01 and 0.32. (2) ΔLGE Model 3: <0.01 and 0.47.

^†^Δ LVEF (%) = chronic LVEF - acute LVEF. Δ LGE scar (%) = chronic LGE - acute LGE.

^‡^The covariates in the models were stepwise forward selected with *p*<0.3. Model 3: Model 2 + further adjustment for the acute phase LV index corresponding to the dependent variable and its interaction with LADSS Group. For the covariates of Models 1 and 2, see **Table 5**.

^§^Statistical significance was set at *p*<0.017 after Bonferroni correction.

LADSS, left atrial diastasis strain slope; LVEF, left ventricular ejection fraction; LGE, late gadolinium enhancement; LA, left atrium; BMI, body mass index.

The bold values represent p<0.05, or p<0.017 after Bonferroni correction for LADSS groups, indicating statistical significance.
